# Brain metastases in Japanese NSCLC patients: prognostic assessment and the use of osimertinib and immune checkpoint inhibitors—retrospective study

**DOI:** 10.1186/s13014-023-02218-3

**Published:** 2023-02-07

**Authors:** Hajime Higaki, Kentaro Nishioka, Manami Otsuka, Noboru Nishikawa, Motoyasu Shido, Hideki Minatogawa, Yukiko Nishikawa, Rikiya Takashina, Takayuki Hashimoto, Norio Katoh, Hiroshi Taguchi, Rumiko Kinoshita, Koichi Yasuda, Takashi Mori, Yusuke Uchinami, Fuki Koizumi, Yoshihiro Fujita, Shuhei Takahashi, Takahiro Hattori, Noriaki Nishiyama, Hidefumi Aoyama

**Affiliations:** 1 Department of Radiation Oncology, Faculty of Medicine and Graduate School of Medicine, Hokkaido University, Kita 15-Jo Nishi 7-Chome, Kita-Ku, Sapporo, Hokkaido 060-8638 Japan; 2 Global Center for Biomedical Science and Engineering, Faculty of Medicine, Hokkaido University, Sapporo, Hokkaido Japan; 3 Department of Radiation Therapy, Hokkaido Cancer Center, Sapporo, Japan; 4 Department of Radiation Oncology, Hokkaido University Hospital, Sapporo, Japan

**Keywords:** DS-GPA, Lung-molGPA, Brain metastasis, Lung cancer, Retrospective study

## Abstract

**Background:**

The Graded Prognostic Assessment for lung cancer using molecular markers (Lung-molGPA) has not been validated for use with Japanese non-small cell lung cancer (NSCLC) patients with brain metastasis (BM) and the factors impacting survival need to be assessed.

**Methods:**

We retrospectively analyzed 294 NSCLC patients who were newly diagnosed with BM between 2013 and 2020 and had received radiotherapy for BM initially at the Hokkaido Cancer Center. We evaluated the effect on the prognosis of Lung-molGPA items, the expression of PD-L1 (classified as high, low, and no expression), and the treatment history. The main outcome was the survival measured from the day of the diagnosis of BM, and log-rank tests were performed to evaluate the results.

**Results:**

The median overall survival (OS) times for adenocarcinoma by groups of GPA scores (0‒1.0, 1.5‒2.0, 2.5‒3.0, and 3.5‒4.0) were 5.5, 14.8, 28.3, and 39.0 months (p < 0.0001), respectively. The median survival times for non-adenocarcinoma by groups of GPA scores (0‒1.0, 1.5‒2.0, and 2.5‒3.0) were 3.2, 11.0, and 16.0 months (p = 0.0011), respectively. In adenocarcinoma patients with gene mutations, osimertinib significantly improved the outcome (median OS: 34.2 and 17.6 months with and without osimertinib, respectively (p = 0.0164)). There was no significant difference in the OS between patients who were initially treated with tyrosine-kinase inhibitor for BM and those who initially received radiotherapy (p = 0.5337). In patients tested for PD-L1 expression, the median survival times after the diagnosis of BM were 5.6, 22.5, and 9.3 months for the high-, low- and no-expression groups (p = 0.2198), respectively. Also, in patients with high PD-L1 expressions, those with ICI had survival (median OS, 8.6 months) than those without (median OS, 3.6 months).

**Conclusions:**

We confirmed that Lung-molGPA successfully classified Japanese NSCLC patients with BM by the prognosis. Osimertinib prolonged survival of EGFR-positive NSCLC patients with BM, and ICI was effective in patients with high PD-L1 expressions.

**Supplementary Information:**

The online version contains supplementary material available at 10.1186/s13014-023-02218-3.

## Background

Symptomatic metastatic brain tumors have been reported to occur in 8–10% of all cancer patients, with lung cancer accounting for half of the primary tumors [1, 2]. The incidence of brain metastases (BM) is on the rise due to the improvement of overall survival time (OS) in patients with carcinomas caused by advances in the treatment of malignant tumors and the development of diagnostic techniques such as high-field MRI that can detect microscopic lesions [3]. The treatment of BM can be broadly classified into surgery, radiotherapy, and chemotherapy, but the most appropriate treatment is unclear given the variety of patient backgrounds, including the number and size of the BM, biological characteristics of each cancer type, general condition, treatment history, and prognosis [4].

Diagnosis-specific graded prognostic assessment (DS-GPA) is a well-established prognostic indicator assessment approach based on clinical data from a group of patients being treated at multiple centers, mainly in North America [5]. The target diseases for this assessment are non-small cell lung cancer (NSCLC), small cell lung cancer, breast cancer, renal cancer, and gastrointestinal tumors [6]. The list has been revised every few years since it was first reported, and DS-GPA for NSCLC was redefined as Lung-molGPA in 2017, with an adjustment for the findings that patients with gene alteration of epidermal growth factor receptor (EGFR) and/or anaplastic lymphoma kinase (ALK) exhibited favorable prognosis [7]. In a previous study of patients with BM treated with radiotherapy, Rice et al. concluded that Lung-molGPA was the best among several prognostic models [8]. Since Lung-molGPA was published, there has been remarkable improvements in the systemic treatment of NSCLC, including the emergence of new EGFR tyrosine-kinase inhibitors (TKI) and immune checkpoint inhibitors (ICI). Osimertinib, a third-generation EGFR-TKI targeting NSCLC with EGFR T790M mutations, is now a first-line treatment for EGFR mutation-positive advanced NSCLC patients, and it has also been shown to demonstrate higher effectiveness for BM than the older EGFR-TKIs including gefitinib or erlotinib [9]. Recent reports have suggested that the order of treatment by TKI and radiotherapy can affect the prognosis [10]. In addition, the expression of programmed death-ligand 1 (PD-L1) and the use of ICI could also have some impact on the prognosis for NSCLC patients with BM [11]. Other than for improvements in systemic agents since its publication, this index has not been verified in Japanese populations; this is a barrier, as patients in East Asia are known to have EGFR mutations significantly more frequently than those of European descent [12].

The purpose of this study was to validate the robustness of Lung-molGPA with Japanese patients, as well as to assess the prognostic impact of the expression of PD-L1 (tumor factor) and the use of osimertinib and ICI (treatment factor).

## Methods

### Patients

We performed a retrospective analysis on 294 NSCLC patients who were newly diagnosed with BM between 2013 and 2020 and who had received radiotherapy for BM initially at the Hokkaido Cancer Center. Exclusion criteria were patients with recurrent BM and/or leptomeningeal metastases.

### Statistics

Survival was measured from the date of diagnosis of BM to the date of death or last follow-up. We evaluated the following Lung-molGPA items: age at diagnosis of BM, Karnofsky Performance Status (KPS), number of BM, presence of extracranial metastasis, and presence of EGFR or ALK mutations (in adenocarcinoma cases only).

We also considered the history of TKI use, especially osimertinib, in the mutation-positive patients. To investigate the correlation of ICI with survival in patients tested for PD-L1, the expression of PD-L1 was classified into three groups: high expression (≥ 50% of cells), low expression (1–49%), and no expression (< 1%), based on the classifications of the clinical treatment strategy.

We used the Kaplan–Meier method to estimate overall survival, and the log-rank test to compare results. To assess the prognostic value of Lung-molGPA items and treatments, univariate analysis, and multiple Cox proportional hazards regression analysis were performed. All P values were two-tailed, and values smaller than 0.05 were considered statistically significant. To compare multiple groups by outcome, the Bonferroni correction was applied. We performed the analysis using JMP Pro (version 16.0.0).

## Results

### Patient characteristics

The detailed patient characteristics are shown in Additional file 1: Supplementary Table. The median follow-up time after diagnosis of BM for all patients was 13.6 months (IQR 5.5‒35.8). The ratio of patients who had extracranial metastases for adenocarcinoma was 65.8%. In adenocarcinoma patients, 41.6% were positive for EGFR and/or ALK mutations. Of all patients, 74 (25.2%) had PD-L1-tests, and the proportions of high, low, and no expression of PD-L1 were very similar among adenocarcinoma and non-adenocarcinoma patients. Most of the patients in whom PD-L1 expression was tested were diagnosed with BM in 2018 or later.

**Fig. 1:**
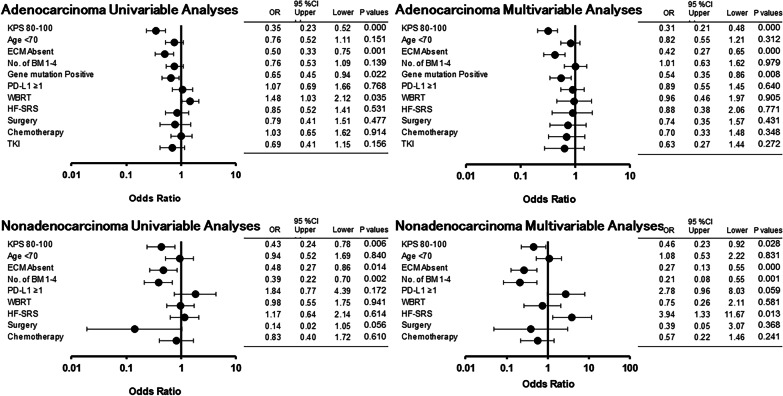
Univariate and multivariate analyses. Univariate and multivariate analyses for adenocarcinoma and non-adenocarcinoma regarding the following items: KPS, age, extracranial metastases, number of brain metastasis, gene mutation, PD-L1 expression, whole brain radiotherapy, hypo-fractionated stereotactic radiosurgery, surgery, chemotherapy and use of a tyrosine-kinase inhibitor

Figure 1 shows the univariate and multivariate analyses of hazard risks for each item. In the intergroup comparison, there was a significant difference (p < 0.05) in risk among the groups about KPS and between the groups with and without extracranial metastasis within the adenocarcinoma and non-adenocarcinoma groups. There was also a significant difference in risk between the groups with and without genetic mutations within the adenocarcinoma group and in risk between groups about the number of BM within non-adenocarcinoma.

### Lung-molGPA

**Fig. 2:**
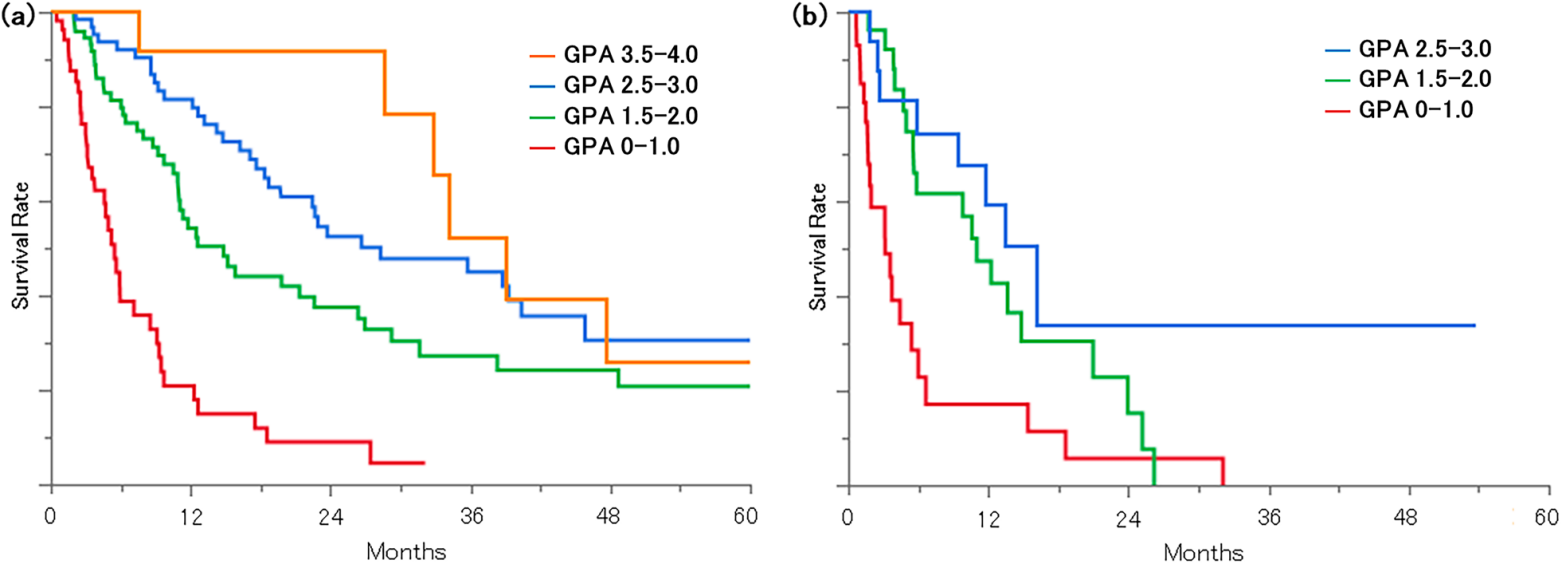
Kaplan–Meier curves for survival from BM diagnosis stratified by Lung-molGPA score. **a** Adenocarcinoma **b** Nonadenocarcinoma

Figure 2 shows the Kaplan–Meier curves for survival by diagnosis and Lung-molGPA. The median survival times for adenocarcinoma groupings by GPA scores of 0‒1.0, 1.5‒2.0, 2.5‒3.0, and 3.5‒4.0 were 5.5, 14.8, 28.3, and 39.0 months (p < 0.0001), respectively. The median survival times of the non-adenocarcinoma groupings by GPA scores of 0‒1.0, 1.5‒2.0, and for the 2.5‒3.0 Group 3.2, 11.0, and 16.0 months (p = 0.0011), respectively.

### Tumor factor

We investigated adenocarcinoma patients with gene mutations for survival time. The median survival time was 23.7 months (IQR 12.5–40.4) in 91 patients (88 EGFR-positive patients and 3 ALK-positive patients).

**Fig. 3:**
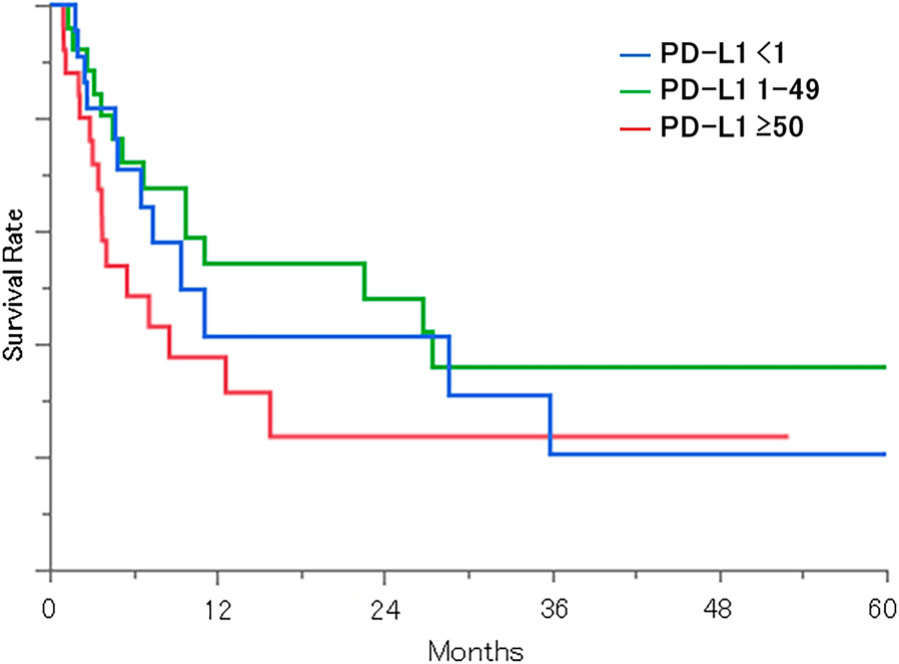
Kaplan–Meier curves for survival from BM diagnosis stratified by PD-L1 expression

Seventy-four patients (62 adenocarcinoma patients and 12 non-adenocarcinoma patients) had been tested for PD-L1 expression. The high- (TPS: ≥ 50), low- (TPS: 1–49), and no-expression groups (TPS: < 1) included 25, 27, and 22 patients, respectively. The median survival times after diagnosis of BM were 5.6 months (IQR 3.1–15.8), 22.5 months (5.2–67.9), and 9.3 months (4.7–35.8) for the high-, low- and no-expression groups (p = 0.2198), respectively (Fig. 3).

### Treatment factor

**Fig. 4:**
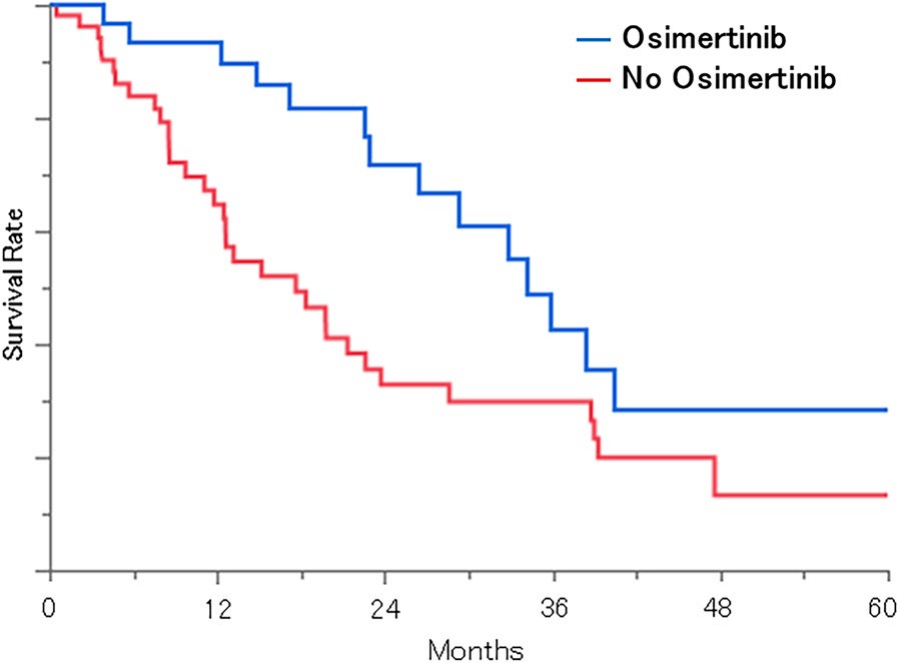
Kaplan–Meier curves for survival from BM diagnosis stratified by the use of Osimertinib

Figure 4 shows the Kapan-Meier curves for survival by diagnosis and the use of osimertinib. 33 patients were treated with osimertinib in 88 EGFR mutation-positive patients. The median survival for the group without osimertinib was 17.6 months (8.6–39.0), while that for the group with osimertinib was 34.2 months (22.9–67.9) (p = 0.0164). There was no significant difference in OS between patients who were initially treated with TKI for BM and those who initially received radiotherapy (p = 0.5337).

Among the 74 patients tested for PD-L1 expression, 42 patients received ICI treatment. And the numbers of these patients in the high-, low- and no-expression groups were 17, 15, and 10, respectively. The types of ICI used were pembrolizumab (27, 64.3%), nivolumab (5, 11.9%), atezolizumab (10, 23.8%), and durvalumab (2, 4.8%) (allowing duplication). The median survivals in the groups treated with and without an ICI were 11.0 months (5.2‒NR) and 9.3 months (3.1‒67.9), respectively (p = 0.4535). In the examination of PD-L1 expression and ICI treatment, ICI improved median survival in patients with high expression [with ICI, 17 patients, median OS 8.6 months (4.1-NR); without ICI, 8 patients, median OS 3.6 months (2.1-NR)]; however, this difference did not reach significance (p = 0.2113).

## Discussion

### Validation of the Lung-molGPA in Japanese patients and tumor factors

In this study, we found that Lung-molGPA can appropriately stratify the prognosis of NSCLC patients with BM, strongly suggesting that Lung-molGPA is applicable to Japanese patients. Similar research has been performed for other racial ancestry patients and others of Asian ancestry [13, 14], but to the best of our knowledge, this is the first such study to be performed for Japanese patients. The proportion of EGFR mutations was markedly higher than in previous studies (235/1521, 15%) [5], which may be due to a trend specific to Asian ancestry patient lung cancers [12]. The median overall survival in each GPA group was not obviously different from those of previous studies. Adenocarcinoma patients had a similar survival to that of a recent database (median 5.5 vs. 7 months; median 14.8 vs. 13 months; median 28.3 vs. 25 months; median 39.0 vs. 46 months); the same held for non-adenocarcinoma patients (median 3.2 vs. 5 months, median 11.0 vs. 10 months, and median 16.0 vs. 13 months) [5].

### Tyrosine-kinase inhibitor

It is generally accepted that there are few effective chemotherapies for BM due to the blood–brain barrier, while EGFR-TKIs, especially osimertinib, can be used for BM of adenocarcinoma. Previous retrospective studies and analyses have suggested that osimertinib is effective for central nervous system lesions in lung cancer and contributes to prolonged OS in non-small cell lung cancers with BM [9]. Our data support the previous report concerning the effectiveness of osimertinib for BM in EGFR mutation-positive patients.

The previous study reported that the order of TKI and radiotherapy affects prognosis in treatment-naïve patients with BM [15]. Although we could not evaluate the effect fully because this study had the small number of patients and included some with a history of treatment; we did not confirm a significant difference in survival between the patients who were initially treated with TKI and those who initially received radiotherapy.

We know that gene mutation is a favorable prognostic factor, and the present analysis confirmed this and suggested that the prognosis may be further refined among patients with genetic mutations.

### Programmed death-ligand 1 expression and immune checkpoint inhibitor use

In this study, PD-L1 expression rates were similar for adenocarcinoma and non-adenocarcinoma, consistent with statistics from previous large clinical trials [16], and there were no significant differences in either PD-L1 expression rates. Among all of the groups, the high expression group had the poorest prognosis, consistent with a meta-analysis on PD-L1 and overall survival [17]. This may be because PD-L1 is essentially a protein that inhibits programmed cell death [18]. Sperduto et al. [19] showed that the PD-L1 status correlated with the prognosis in a large retrospective study, but our results disagree with this. One reason may be that the number of cases in our study was not sufficiently large. It is also possible that ICI was not yet common during the time when the patients in this study were treated. In other words, a high PD-L1 expression may not be a favorable prognostic factor in settings where ICI is not commonly used.

### Limitations

This study is a single-center, retrospective analysis, and the target population is subject to selection bias. The results do not clearly indicate an optimal treatment for BM. Also, patients who did not receive radiation therapy for BM were not included in the study, and this means that patients who responded well to drug therapy or did not have the opportunity to go to the hospital were not included. In other words, the study may not adequately reflect the survival of the entire population with BM.

## Conclusions

Our findings suggest that Lung-molGPA is applicable to Japanese patients. Osimertinib may be more effective for NSCLC patients with gene mutation than other TKI though the prognostic value of PD-L1 status remains to be investigated in a larger number of patients. Prospective trials should be scheduled so that Lung-molGPA can be used to determine treatment strategy.

## Supplementary Information

**Additional file 1**. Patient characteristics and median survival. NSCLC, non-small cell lung carcinoma; MS, median survival; IQR, interquartile range; KPS, Karnofsky performance status; BM, brain metastasis; ECM, extracranial metastases; EGFR, epidermal growth factor receptor; ALK, anaplastic lymphoma kinase; WBRT, whole-brain radiotherapy; HF-SRS, hypo-fractionated stereotactic radiosurgery; PD-L1, programmed death-ligand 1; TPS, tumor proportion score; NR, not reached

## Data Availability

The datasets analysed during the current study are available from the corresponding author on reasonable request.

## Abbreviations

DS-GPA: Diagnosis-specific graded prognostic assessment
NSCLC: Non-small cell lung cancer
BM: Brain metastasis
PD-L1: Programmed death-ligand 1
ICI: Immune checkpoint inhibitors
OS: Overall survival
EGFR: Epidermal growth factor receptor
ALK: Anaplastic lymphoma kinase
TKI: Tyrosine-kinase inhibitor
KPS: Karnofsky performance status
WBI: Whole-brain irradiation
HF-SRS: Hypo-fractionated stereotactic radiosurgery

## References

[1] Schouten LJ, Rutten J, Huveneers HAM, Twijnstra A (2002). Incidence of brain metastases in a cohort of patients with carcinoma of the breast, colon, kidney, and lung and melanoma. Cancer.

[2] Barnholtz-Sloan JS, Sloan AE, Davis FG, Vigneau FD, Lai P, Sawaya RE (2004). Incidence proportions of brain metastases in patients diagnosed (1973 to 2001) in the Metropolitan Detroit Cancer Surveillance System. J Clin Oncol.

[3] Carden CP, Agarwal R, Saran F, Judson IR. Personal view eligibility of patients with brain metastases for phase I trials: Time for a rethink? [Internet]. Vol. 9, www.thelancet.com/oncology. 2008. Available from: www.thelancet.com/oncology 10.1016/S1470-2045(08)70257-2 19071257

[4] Aoyama H, Tago M, Shirato H (2015). Stereotactic radiosurgery with or without whole-brain radiotherapy for brain metastases: secondary analysis of the JROSG 99-1 randomized clinical trial. JAMA Oncol.

[5] Sperduto PW, Mesko S, Li J, Cagney D, Aizer A, Lin NU (2020). Survival in patients with brain metastases: summary report on the updated diagnosis-specific graded prognostic assessment and definition of the eligibility quotient. J Clin Oncol.

[6] Villano JL, Durbin EB, Normandeau C, Thakkar JP, Moirangthem V, Davis FG (2015). Incidence of brain metastasis at initial presentation of lung cancer. Neuro Oncol.

[7] Sperduto PW, Yang TJ, Beal K, Pan H, Brown PD, Bangdiwala A (2017). Estimating survival in patients with lung cancer and brain metastases an update of the graded prognostic assessment for lung cancer using molecular markers (Lung-molGPA). JAMA Oncol.

[8] Rice SR, Bentzen SM, Hanna A, Choi E, Boggs DH, Kwok Y (2018). Prognostic models for patients with brain metastases after stereotactic radiosurgery with or without whole brain radiotherapy: a validation study. J Neurooncol.

[9] Wu YL, Ahn MJ, Garassino MC, Han JY, Katakami N, Kim HR (2018). CNS efficacy of osimertinib in patients with T790M-positive advanced non-small-cell lung cancer: data from a randomized phase III trial (AURA3). J Clin Oncol.

[10] Fan KY, Lalani N, LeVasseur N, Krauze A, Hsu F, Gondara L (2021). Type and timing of systemic therapy use predict overall survival for patients with brain metastases treated with radiation therapy. J Neurooncol.

[11] Goldberg SB, Gettinger SN, Mahajan A, Chiang AC, Herbst RS, Sznol M (2016). Pembrolizumab for patients with melanoma or non-small-cell lung cancer and untreated brain metastases: early analysis of a non-randomised, open-label, phase 2 trial. Lancet Oncol.

[12] Kohno T, Nakaoku T, Tsuta K, Tsuchihara K, Matsumoto S, Yoh K (2015). Beyond ALK-RET, ROS1 and other oncogene fusions in lung cancer. Transl Lung Cancer Res.

[13] Nieder C, Hintz M, Oehlke O, Bilger A, Grosu AL (2017). Validation of the graded prognostic assessment for lung cancer with brain metastases using molecular markers (lung-molGPA). Radiat Oncol.

[14] Li J, Jing W, Zhai X, Jia W, Zhu H, Yu J (2021). Estimating survival in patients with non-small-cell lung cancer and brain metastases: a verification of the graded prognostic assessment for lung cancer using molecular markers (lung-molgpa). Onco Targets Ther.

[15] William JM, Lester-Coll NH, Wu AJ, Yang TJ, Lockney NA, Gerber NK (2017). Management of brain metastases in tyrosine kinaseinhibitor–na ¨ive epidermal growth factor receptor–mutantnon–small-cell lung cancer: a retrospective multi-institutional analysis. J Clin Oncol Am Soc Clin Oncol.

[16] Mok TSK, Wu YL, Kudaba I, Kowalski DM, Cho BC, Turna HZ (2019). Pembrolizumab versus chemotherapy for previously untreated, PD-L1-expressing, locally advanced or metastatic non-small-cell lung cancer (KEYNOTE-042): a randomised, open-label, controlled, phase 3 trial. The Lancet.

[17] Pan ZK, Ye F, Wu X, An HX, Wu JX (2015). Clinicopathological and prognostic significance of programmed cell death ligand1 (PD-L1) expression in patients with non-small cell lung cancer: a meta-analysis. J Thorac Dis.

[18] Brody R, Zhang Y, Ballas M, Siddiqui MK, Gupta P, Barker C, et al. PD-L1 expression in advanced NSCLC: insights into risk stratification and treatment selection from a systematic literature review. In: Lung cancer. Vol. 112, Elsevier Ireland Ltd; 2017. p. 200–15. 10.1016/j.lungcan.2017.08.005 29191596

[19] Sperduto PW, De B, Li J, Carpenter D, Kirkpatrick J, Milligan M (2022). Graded prognostic assessment (GPA) for patients with lung cancer and brain metastases: initial report of the small cell lung cancer GPA and update of the non-small cell lung cancer GPA including the effect of programmed death ligand 1 and other prognostic factors. Int J Radiat Oncol Biol Phys.

