# Supplementary material for: Brain metastases in Japanese NSCLC patients: prognostic assessment and the use of osimertinib and immune checkpoint inhibitors—retrospective study
Source: Radiat Oncol. 2023 Feb 7;18:25. doi: 10.1186/s13014-023-02218-3 (PMC9903535; doi:10.1186/s13014-023-02218-3)
Supplement: Supplementary file 1 — Additional file 1. Patient characteristics and median survival. NSCLC, non-small cell lung carcinoma; MS, median survival; IQR, interquartile range; KPS, Karnofsky performance status; BM, brain metastasis; ECM, extracranial metastases; EGFR, epidermal growth factor receptor; ALK, anaplastic lymphoma kinase; WBRT, whole-brain radiotherapy; HF-SRS, hypo-fractionated stereotactic radiosurgery; PD-L1, programmed death-ligand 1; TPS, tumor proportion score; NR, not reached [file 13014_2023_2218_MOESM1_ESM.docx]

|  | NSCLC Adenocarcinoma | | NSCLC Non-adenocarcinoma | |
| --- | --- | --- | --- | --- |
|  | No. (%) | MS (IQR) | No. (%) | MS (IQR) |
| Overall | 219 | 17.5 (7.1-40.4) | 75 | 6.6 (3.2-18.6) |
| KPS at BM diagnosis | | | | |
| 90-100 | 98 (44.7) | 28.6 (11.3-67.9) | 27 (36.0) | 13.4 (5.6-26.2) |
| 80 | 62 (28.3) | 12.6 (7.1-48.7) | 21 (28.0) | 6.6 (5.0-23.9) |
| ≤70 | 59 (26.9) | 5.9 (3.7-19.8) | 27 (36.0) | 3.7 (1.7-9.8) |
| Age at BM diagnosis (Year) | | | | |
| <70 | 133 (60.7) | 19.8 (7.5-48.7) | 30 (40.0) | 6.0 (3.6-16.0) |
| ≥70 | 86 (39.3) | 13.2 (5.5-34.2) | 45 (60.0) | 10.6 (3.2-18.6) |
| ECM at BM diagnosis | | | | |
| Absent | 75 (34.2) | 31.6 (12.2-67.9) | 38 (50.7) | 12.2 (5.0-23.9) |
| Present | 144 (65.8) | 12.3 (5.5-29.2) | 37 (49.3) | 4.7 (1.7-14.8) |
| No. of BM at initial diagnosis | | | | |
| 1-4 | 110 (50.2) | 22.7 (7.9-47.6) | 45 (60.0) | 12.2 (5.6-23.9) |
| ≥5 | 109 (49.8) | 15.8 (6.0-35.8) | 30 (40.0) | 3.9 (1.9-6.0) |
| Gene mutation (EGFR / ALK) | | | | |
| Positive | 91 (41.6) | 23.7 (12.5-40.4) |  |  |
| Negative / Unknown | 128 (58.4) | 10.5 (4.6-45.8) |  |  |
| WBRT included in initial therapy | | | | |
| Yes | 117 (53.4) | 12.6 (3.9-38.3) | 41 (54.7) | 5.4 (3.2-21.0) |
| No | 102 (46.6) | 19.8 (10.5-47.6) | 34 (45.3) | 9.8 (4.7-14.8) |
| HF-SRS included in initial therapy | | | | |
| Yes | 35 (16.0) | 16.2 (7.3-NR) | 24 (32.0) | 9.8 (3.1-14.8) |
| No | 184 (84.0) | 17.5 (6.4-39.3) | 51 (68.0) | 6.0 (3.6-21.0) |
| Surgery included in initial therapy | | | | |
| Yes | 21 (9.6) | 18.7 (9.7-NR) | 6 (8.0) | NR (NR-NR) |
| No | 198 (90.4) | 16.2 (7.1-39.3) | 69 (92.0) | 6.0 (3.2-15.4) |
| Chemotherapy used as initial therapy | | | | |
| Yes | 36 (16.4) | 14.8 (8.6-28.6) | 12 (16.0) | 11.0 (5.6-21.0) |
| No | 183 (83.6) | 18.3 (6.0-45.8) | 63 (84.0) | 5.9 (2.7-16.0) |
| Tyrosine-kinase Inhibitor used as initial therapy | | | | |
| Yes | 29 (13.2) | 22.5 (11.8-67.9) |  |  |
| No | 190 (86.8) | 15.8 (5.9-39.3) |  |  |
| PD-L1(TPS) | | | | |
| ≥50 | 21 (9.6) | 4.1 (3.1-15.8) | 4 (5.3) | 5.6 (3.3-NR) |
| 1-49 | 23 (10.5) | 27.4 (9.7-67.9) | 4 (5.3) | 2.9 (2.0-4.9) |
| <1 | 18 (8.2) | 11.0 (6.4-35.8) | 4 (5.3) | 4.7 (3.2-NR) |
| Untested | 157 (71.7) | 18.3 (8.6-39.3) | 63 (84.0) | 10.6 (3.7-18.6) |
| Immune Checkpoint Inhibitor use | | | | |
| Yes | 54 (24.7) | 18.3 (8.6-45.8) | 15 (20.0) | 5.6 (4.4-16.0) |
| No | 165 (75.3) | 15.2 (6.0-39.0) | 60 (80.0) | 9.8 (3.1-21.0) |
| Osimertinib use | | | | |
| Yes | 33 (15.1) | 34.2 (22.9-67.9) |  |  |
| No | 186 (84.9) | 12.6 (5.9-39.3) |  |  |
| Lung-GPA | | | | |
| 0-1.0 | 56 (25.6) | 5.5 (3.0-9.5) | 29 (38.7) | 3.2 (1.7-6.0) |
| 1.5-2.0 | 82 (37.4) | 14.8 (7.4-38.3) | 29 (38.7) | 11.0 (5.0-21.0) |
| 2.5-3.0 | 67 (30.6) | 28.3 (14.2-NR) | 17 (22.7) | 16.0 (5.9-NR) |
| 3.5-4.0 | 14 (6.4) | 39.0 (32.8-67.9) |  |  |
